# Citrate Synthase Insufficiency Leads to Specific Metabolic Adaptations in the Heart and Skeletal Muscles Upon Low-Carbohydrate Diet Feeding in Mice

**DOI:** 10.3389/fnut.2022.925908

**Published:** 2022-07-07

**Authors:** Kanako Sumi, Yuiko Hatanaka, Reina Takahashi, Naoko Wada, Chihiro Ono, Yuri Sakamoto, Hirohito Sone, Kaoruko Iida

**Affiliations:** ^1^Department of Food and Nutrition Science, Graduate School of Humanities and Sciences, Ochanomizu University, Bunkyo, Japan; ^2^Department of Clinical Dietetics and Human Nutrition, Faculty of Pharmacy and Pharmaceutical Sciences, Josai University, Sakado, Japan; ^3^Department of Hematology, Endocrinology and Metabolism, Faculty of Medicine, Niigata University, Niigata, Japan; ^4^Department of Endocrinology and Metabolism, Faculty of Medicine, University of Tsukuba, Tsukuba, Japan; ^5^The Institute for Human Life Innovation, Ochanomizu University, Bunkyo, Japan

**Keywords:** citrate synthase, heart, knockout mice, skeletal muscle, TCA cycle

## Abstract

A decrease in TCA cycle activity may lead to impaired nutrition metabolism and cellular energy shortage. Herein, we aimed to characterize the detailed metabolic changes that compensate for energy shortages in energy-consuming organs (heart and skeletal muscles) in mice with knockout of citrate synthase (CS), an important enzyme in the TCA cycle. CS hetero knockout (CS +/−) mice and wild-type mice were fed a low-carbohydrate ketogenic diet (LCKD) or high-fat, high-carbohydrate diet (HFHCD) to induce metabolic changes. Body weight, blood serum parameters, metabolic gene expression, and adenosine triphosphate (ATP) levels were measured in the heart and skeletal muscles. Glycogen content, anabolic and catabolic biomarkers, and morphological changes were also assessed in the skeletal muscles. After diet feeding, there were no differences observed in the body weight and blood serum parameters between wild-type and CS +/− mice. The cardiac expression of genes related to the utilization of fatty acids, monocarboxylates, and branched amino acids increased in LCKD-fed CS +/− mice. In contrast, no significant differences in gene expression were observed in the muscles of LCKD-fed mice or the heart and muscles of HFHCD-fed mice. ATP levels decreased only in the skeletal muscles of LCKD-fed CS +/− mice. Additionally, the decrease in glycogen content, suppression of p70 S6 kinase, and presence of type I fiber atrophy were observed in the muscles of LCKD-fed CS +/− mice. These results suggest that the energy-consuming organs with CS insufficiency may undergo tissue-specific adaption to compensate for energy shortages when the carbohydrate supply is limited.

## Introduction

The production of adenosine triphosphate (ATP) is essential for organ function, particularly for the mechanical and electrical functions of high energy-consuming organs such as the heart and skeletal muscles (1). In most cells, the mitochondrial tricarboxylic acid (TCA) cycle acts as the central driver of ATP biosynthesis. In eukaryotes, the TCA cycle operates in the matrix of the mitochondria following the biosynthesis of acetyl-CoA *via* the oxidation of mainly fat and carbohydrates (2, 3). Although fat is the main substrate for cardiac ATP production, the heart can also use other energy sources through multiple mechanisms, such as the control of enzyme activity, signal transduction events, and gene regulation (4, 5). In contrast, the skeletal muscles use glucose as their main fuel source and can switch to the use of fatty acids (FAs) under certain conditions (6).

Aging and metabolic diseases such as type 2 diabetes are closely associated with a decrease in the mitochondrial capacity including the TCA cycle activities (7–10), which could lead to the disruption of nutrient metabolism and a shortage of cellular energy. Recent studies have reported that the dysfunction of the TCA cycle along with a loss or a decrease in the activity of its related enzymes is involved in the etiology of various diseases (11). However, the effects of such TCA cycle impairment on the flexibility and capacity of substrate metabolism in the heart and skeletal muscles have not yet been thoroughly investigated. Tissue-specific adaptive responses to the impairments of this pathway should be determined to understand the mechanisms involved in the development of geriatric changes and metabolic disorders.

Citrate synthase (CS) is the rate-limiting enzyme of TCA cycle and plays a pivotal in regulating energy production through mitochondrial respiration. CS converts acetyl-CoA and oxaloacetate into citrate, and its activity is sometimes used as a biomarker of mitochondrial content and function (12, 13). CS activity in the muscles is increased by physical activities (14) and decreases with aging (15, 16) or in metabolic disorders such as type 2 diabetes (17). Animal studies revealed that in aged hearts, the gene expression and catalytic activity of CS were significantly decreased among TCA cycle enzymes (18, 19). Therefore, we considered CS knockout mice to be useful to investigate the effects of TCA cycle dysfunction on metabolic adaptations of the heart and skeletal muscles.

Homozygote CS knockout (CS–/–) mice present with embryonic lethality; therefore, heterozygous CS knockout (CS +/−) mice are preferred. The gene and protein expression and activity of CS in CS–/+ mice are approximately half of those in their wild-type (WT) (+/+) siblings. However, unexpectedly, we found that there were almost no differences in the growth, morphology, blood parameters, insulin sensitivity, ATP levels, and metabolic gene expression in the organs (including in the heart and skeletal muscles) between WT and CS +/− mice fed a regular diet (20). In contrast, previous reports showed that the H55N polymorphism in the mouse *Cs* gene was linked to low enzyme activity and the mice having this polymorphism exhibited decreased glucose tolerance during high-fat diet feeding (21, 22). Thus, we predicted that metabolic differences could be observed between WT and CS +/− mice under specific nutritional conditions.

Thus, we aimed to examine whether CS insufficiency affects the metabolic response of the heart and skeletal muscles in mice fed a special diet: a low-carbohydrate ketogenic diet (LCKD) that mimics a fasting state or a high-fat, high-carbohydrate (HFHCD) obesogenic diet that induces glucose intolerance. In this study, we investigated how chronic impairment of the mitochondrial CS activity affects the adaptive metabolic response to a ketogenic or obesogenic environment. Decrease in CS activity may cause energy deficiency, forcing the cell to rely on the most primitive route of energy production: cytosolic glycolysis. However, LCKD contains almost no glucose, which is the only source of the glycolytic pathway, and HFHCD induces insulin resistance (23), which disrupts glucose uptake. Therefore, it is interesting to assess how CS-insufficient organisms control their metabolism when consuming an LCKD or HFHCD.

## Materials and Methods

### Animals and Rearing Conditions

Heterozygous CS + ⁣/⁣− founder mice were created at Lexicon Genetics from their OmniBank library of knockout embryonic stem cell clones. We crossed this CS + ⁣/⁣− founder mice more than ten times to transfer the null mutation onto the C57BL6/J genetic background. We used only male mice for the present studies. Male CS + ⁣/⁣− mice and WT littermates at 8–11 weeks of age were used in this study. The mice were housed with 2–4 animals per cage in a temperature- and humidity-controlled facility with a 12-h light/dark cycle and free access to food and water.

### Experimental Design and Diets

In the first experiment, the CS +/− (*n* = 7) and WT (*n* = 7) mice were fed a low-carbohydrate diet (88% fat, 11% protein, and 1% carbohydrate; 7.2 kcal/g), also called ketogenic diet because it induced ketosis in the subject consuming it, for 8 weeks. In the second experiment, the CS +/− (*n* = 7) and WT mice (*n* = 10) were fed a high-fat, high-carbohydrate (42% fat, 18% protein, and 40% carbohydrate; 4.7 kcal/g) for 8 weeks to induce obesity. The ingredients of each diet were purchased from Oriental Yeast Co. (Tokyo, Japan), and the composition of each diet is shown in Supplementary Table 1. Apart from these two main experiments, WT mice (10 weeks old, *n* = 6) were reared on a normal rodent diet (AIN-93G, Oriental Yeast Co.) for 8 weeks to confirm whether each experimental diet was effective at inducing metabolic changes.

The body weight of the mice was measured every 2 weeks throughout the experimental period. At the end of the experimental period, the mice were fasted for 4 h and anesthetized with isoflurane to collect blood samples into 1.5 mL microcentrifuge tubes. The mice were euthanized by cervical dislocation under anesthesia and immediately dissected to collect tissue samples from the heart and hindlimb muscles. All animal procedures were approved by the Animal Ethics Committee of Ochanomizu University (approval number: 19013 and 20018).

### Biochemical Measurements of Blood Serum

The blood samples were centrifuged at 10,000 × *g* for 5 min in a microcentrifuge (Model 3500, Kubota, Tokyo, Japan), and the supernatant was transferred to a new tube and stored at –20°C until analysis. Blood glucose and non-esterified FA levels were measured using the biochemical colorimetric assay kits LabAssay™ Glucose (Cat. #298-65701, Wako, Osaka, Japan), LabAssay™ Triglyceride (Cat. #290-63701, Wako) and LabAssay™ NEFA (Cat. #294-63601, Wako) according to the manufacturer’s protocols. The ketone body (3-hydroxybutyrate) concentration was measured using a Ketone Test Sanwa kit (Cat. #877434, Sanwa Kagaku, Tokyo, Japan), following the enzymatic protocol. Insulin concentration was evaluated using a Mouse Insulin ELISA kit (Morinaga, Yokohama, Japan), according to the manufacturer’s instructions. All absorbance measurements were performed using an EnSpire^®^ microplate reader (PerkinElmer, Waltham, MA, United States).

### Gene Expression Profiling by Quantitative RT-PCR

The total mRNA was extracted from snap-frozen tissue using Sepasol RNA I reagent (Nacalai Tesque, Kyoto, Japan), according to the manufacturer’s protocol. The RNA was treated with DNase and reverse-transcribed using ReverTra Ace™ qPCR RT Master Mix (Toyobo, Osaka, Japan). Diluted cDNA was used as a template to quantify the relative mRNA concentration. SYBR^®^ Premix Ex Taq™ (Takara Bio, Shiga, Japan) was used to prepare the quantitative RT-PCR mixtures, which were evaluated on a Thermal Cycler Dice^®^ Real Time System (Takara Bio), according to the manufacturer’s instructions. The relative gene expression values were normalized with that of the β-actin gene. The sequences of the primers used to amplify each gene are shown in Supplementary Table 2.

### Measurement of Adenosine Triphosphate Content

The ATP levels in each tissue were measured using a Tissue ATP assay kit (Toyo B-Net Co., Tokyo, Japan). Briefly, frozen tissues were weighed using a microanalytical balance, homogenized with 10 mL homogenate buffer (0.25 M sucrose, 10 mM HEPES-NaOH, pH 7.4) on ice and centrifuged at 1,000 × *g* for 10 min at 4°C. The supernatant was diluted 1:8 in ATP extraction reagent, left to stand for extraction (30 min), and then used for ATP measurement. ATP levels were quantified based on the luciferin and luciferase chemiluminescence measured in an EnSpire^®^ microplate reader (PerkinElmer) according to the manufacturer’s protocol and expressed per mg of tissue weight.

### Quantification of Glycogen in the Muscle Tissue

Glycogen levels in the muscles collected from the mice were measured using a method based on that of Chan et al. (24). Briefly, the tissues were weighed using a microanalytical balance, dissolved in 30% KOH at 95°C, 2% Na_2_SO_4_, and 66% ethanol were then added, and the final solution was centrifuged (13,000 × *g*, 5 min at 4°C) to obtain a pellet. The pellet was washed twice with 66% ethanol, 0.2 M acetate buffer (pH 4.5) and amyloglucosidase (Sigma-Aldrich, Tokyo, Japan) were added, and the solution was incubated at 37°C for 30 min. The glucose content was measured using the LabAssay™ Glucose kit (Wako, Tokyo, Japan) and the amount of glycogen per gram of tissue (wet weight) was calculated.

### Western Blotting Assays

Tissue samples of the gastrocnemius muscle were crushed in liquid nitrogen and homogenized in lysis buffer containing a protease inhibitor cocktail (Sigma-Aldrich, St. Louis, MO, United States). After centrifugation at 14,000 × *g* for 20 min at 4°C, the supernatant was aliquoted and stored at –20°C. The samples containing 40 μg of total proteins were loaded onto a 10% SDS-PAGE gel, separated using Tris-glycine buffer containing 0.1% SDS, and transferred onto polyvinylidene fluoride membranes (GE Healthcare, Uppsala, Sweden).

The membranes were incubated overnight at 4°C in Blocking One-P solution (Nacalai Tesque, Kyoto Japan) with the following antibodies: AMPK (1:1,000, #2532), phospho-AMPK (1:1,000, #2535), Akt (1:1,000, #9272), phosphor-Akt (Ser473) (1:1,000, #9271), S6K (1:1,000, #9202), phosphor-S6K (1:250, #9205), and LC3B (1:500, #2775) (all from Cell Signaling Technology, Danvers, MA, United States). The membranes were then incubated with a secondary anti-rabbit IgG antibody (1:7,000, Cell Signaling Technology; #7074) in Tris-buffered saline containing 0.1% Tween 20 for 1 h at room temperature (20–25°C). The blots were developed using ECL™ prime western blotting detection reagent (GE Healthcare), and images were captured with an ImageQuant™ LAS 4000 (GE Healthcare) detection system. Normalization was performed using an anti-β-actin antibody (1:1,000, sc-47778; Santa Cruz Biotechnology, Dallas, TX, United States) and a corresponding secondary anti-mouse IgG antibody (1:7,000, #7076; Cell Signaling Technology).

### Histological Analysis

Morphological changes in the muscles of LCKD-fed animals were examined. Transverse cryosections of the gastrocnemius muscles were used to assess the general tissue architecture and measure the cross-sectional area (CSA) of each muscle fiber.

Muscle sections (8 μm thick) were fixed with 10% formalin and then stained with hematoxylin and eosin. For immunofluorescence staining, the sections were air-dried for 10 min and then incubated with blocking solution [phosphate-buffered saline containing 0.1% Tween 20 (PBS-T), 5% bovine serum albumin (BSA), and 0.1% fish gelatin] for 1 h at room temperature. The sections were incubated overnight at 4°C with the mouse monoclonal antibodies directed to the anti-myosin heavy chain, BA-F8 (1:100) specific for myosin heavy chain I predominant in type I fiber or SC-71 (1:100) specific for myosin heavy chain II predominant in type II fiber (Development Studies Hybridoma Bank, Iowa City, IA, United States), diluted in PBS-T containing 0.8% BSA and 0.1% fish gelatin. After washing three times with PBS-T, the sections were incubated for 2 h at room temperature with the following secondary antibodies: FITC-conjugated anti-mouse IgG antibody (1:250, Thermo Fisher Scientific, Waltham, MA, United States) for BA-F8 or rhodamine (TRITC)-conjugated anti-mouse IgG antibody (1:200, Jackson ImmunoResearch, West Grove, PA, United States) for SC-71. After washing three times with PBS-T, the sections were mounted with Fluoromount-G^®^ (SouthernBiotech, Birmingham, AL, United States). For each staining technique, 12 representative fields per animal (four fields from three sections) were chosen, and the myofiber areas in the samples were analyzed using a digital microscope (BZ-X700; Keyence, Osaka, Japan) with its associated software.

### Statistical Analyses

The data were expressed as the mean ± standard error of mean (SEM). All statistical analyses were performed using SPSS Statistics for Windows software (version 24, SPSS, Inc., Chicago, IL, United States). The normality of the variables was assessed using the Shapiro–Wilk test. Unpaired Student’s *t*-test was used to identify significant differences between two groups. Two-way repeated analysis of variance was used to evaluate the differences in body weight between the groups over time. A non-parametric test (Mann–Whitney *U*-test) was used to compare the fiber areas in the muscle samples. Statistical significance was set at *p* < 0.05.

## Results

### Body Weight and Blood Parameters of Citrate Synthase +/− and Wild-Type Mice

**FIGURE 1:**
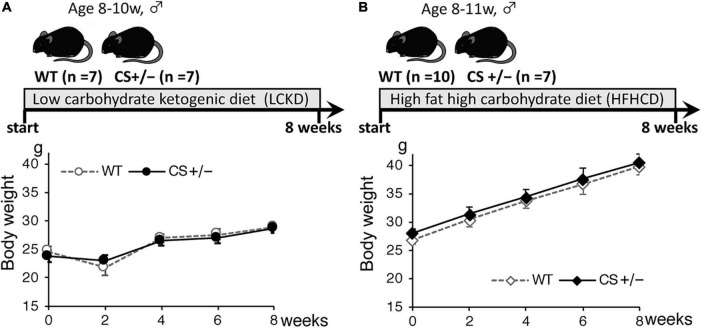
Timeline of the experimental protocol (upper panel) and body weight of mice (lower panel) during the 8-week experimental period. Citrate synthase hetero knockout mice (CS +/−) and their wild-type siblings (WT) were fed **(A)** a low-carbohydrate ketogenic diet (LCKD), or **(B)** a high-fat, high-carbohydrate diet (HFHCD). Data are shown as the mean ± SEM (*n* = 7–10 per group).

The experimental design and body weight of mice during the study are shown in Figure 1. There were no significant differences in body weight between WT and CS +/− mice on both LCKD and HCHFD feeding. The body weight of the LCKD-fed mice decreased after 2 weeks, after which the mice gained weight as they grew. In contrast, all mice fed a HCHFD gained weight throughout the 8-week experimental period (Figure 1). After 8 weeks, the average body weight of LCKD-fed mice was 28.9 ± 1.0 g in the WT group and 28.7 ± 0.8 g in the CS +/− group, and that of HFHCD-fed mice was 39.8 ± 1.1 g for WT mice and 40.5 ± 1.5 g for CS +/− mice (Table 1). Similarly, there were no significant differences in blood glucose, insulin, triglyceride, non-esterified FA, or 3-hydroxybutyrate levels between WT and CS +/− mice in both experiments (Table 1).

**TABLE 1:** Body weight and blood parameters.

	LCKD		HFHCD	
	WT	CS +/−	WT	CS +/−
Initial body weight [g]	24.6 ± 0.9	23.8 ± 1.0	26.9 ± 0.4	28.1 ± 0.7
Final body weight [g]	28.9 ± 1.0	28.7 ± 0.8	39.8 ± 1.1	40.5 ± 1.5
Glucose [mg/dL]	234.3 ± 13.4	204.9 ± 14.4	299.0 ± 14.9	268.9 ± 11.2
Insulin [ng/ml]	0.86 ± 0.16	1.38 ± 0.37	3.86 ± 1.34	3.69 ± 0.81
Triglyceride [mg/ml]	43.6 ± 2.5	45.0 ± 6.6	51.2 ± 3.2	56.9 ± 4.9
NEFA [mEq/L]	1.05 ± 0.08	1.12 ± 0.11	0.63 ± 0.08	0.80 ± 0.08
3-HB [μmol/L]	1387.4 ± 292.5	1320.5 ± 265.6	235.9 ± 16.3	211.2 ± 24.9

*LCKD, low-carbohydrate ketogenic diet; HFHCD, high-fat high-carbohydrate diet; WT, wild type mice; CS +/−, citrate synthase-knockout mice; NEFA, non-esterified fatty acid; 3-HB, 3-hydroxybutyrate; n.d., not determined. The data are shown as mean ± SEM.*

The results of comparison with the reference WT mice fed a regular diet are shown in Supplementary Figure 1. Compared with that of WT mice fed a regular diet, the body weight of HFHCD-fed mice significantly increased, whereas the body weight changes did not differ in LCKD-fed mice during the 8 weeks of experiment. In the blood parameters, the levels of serum 3-hydroxybutyrate and non-esterified FA in LCKD-fed mice and the levels of serum glucose and triglyceride in HCHFD-fed WT mice were significantly higher than those in the reference mice fed a regular diet. These data indicate that both the experimental diets were effective at inducing metabolic changes.

### Expression Profile of Metabolic Genes

**FIGURE 2:**
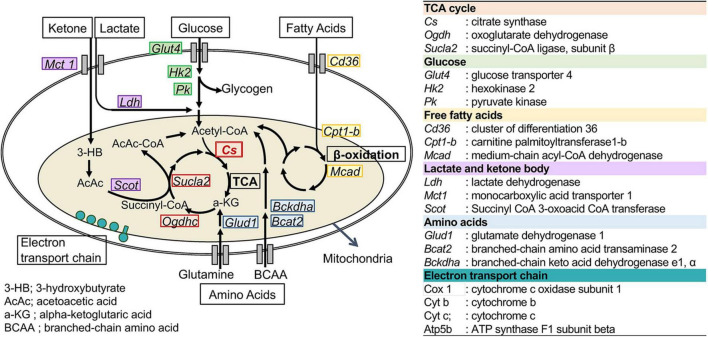
Metabolic genes analyzed in this study. The names and abbreviations of the genes and roles of their encoding proteins in fuel metabolism are summarized.

**FIGURE 3:**
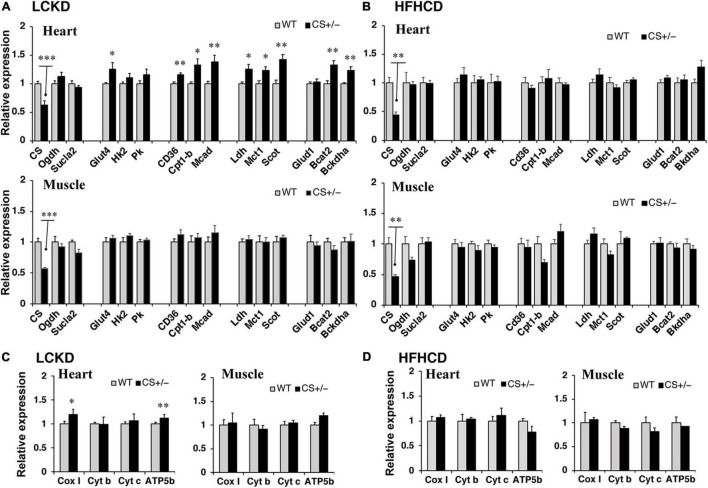
Effects of each diet exposure on metabolic gene expression in the heart and gastrocnemius muscles of citrate synthase-knockout mice (CS +/−, black bars) and their wild-type siblings (WT, gray bars). The mRNA abundance was quantified and normalized to that of β-actin in **(A,C)** mice fed a LCKD and **(B,D)** mice fed a HFHCKD. Values are expressed as the fold-change compared with expression levels in WT mice, which were arbitrarily set to 1 (*p < 0.05; ^**^p < 0.01; ^***^p < 0.001 vs. WT).

To assess the changes in metabolic fuel selection, the expression levels of genes related to substrate utilization in the heart and gastrocnemius muscles at the end of the 8-week experiment were compared between WT and CS +/− mice using quantitative RT-PCR. The tested genes and roles of their encoded proteins in substrate metabolism are summarized in Figure 2. In both the heart and gastrocnemius muscles, *Cs* mRNA expression was significantly lower in CS +/− mice than that in WT mice, as expected (Figure 3). In LCKD-fed mice, significant increases in the expression of genes related to utilization of various substrates, such as FAs, glucose, lactate, ketone bodies, and amino acids, were observed in the heart of CS +/− mice as compared to that in WT mice; however, there were no significant differences in the expression of these genes in the gastrocnemius muscles (Figure 3A). Among the genes related to mitochondrial electron transport chain, the gene expression of cytochrome c oxidase 1 (*Cox1*) encoded on mitochondrial DNA and ATP synthase F1 subunit beta (*Atp-5b*) encoded on genomic DNA were also upregulated in the heart but not in the muscles of LCKD-fed mice (Figure 3C). In contrast, in HFHCD-fed mice, the expression of only the *Cs* gene was significantly different between CS +/− and WT mice in both the heart and gastrocnemius muscles (Figures 3B,D).

**FIGURE 4:**
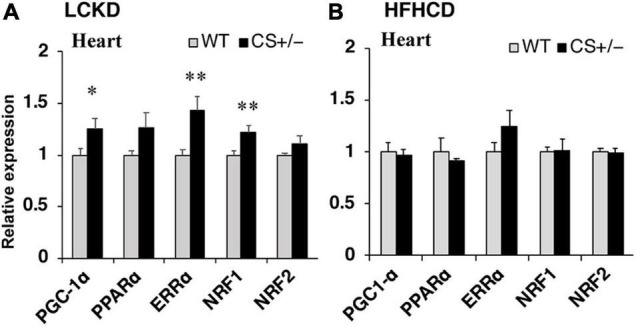
Effects of each diet exposure on the gene expression of transcription factors and transcriptional co-activators related to fuel metabolism in the hearts of citrate synthase-knockout mice (CS +/−, black bars) and their wild-type siblings (WT, gray bars). The mRNA abundance was quantified and normalized to that of β-actin in **(A)** mice fed a LCKD and **(B)** mice fed a HFHCKD. Values are expressed as the fold-change compared with the expression levels in WT mice, arbitrarily set to 1 (*p < 0.05; ^**^p < 0.01 vs. WT).

Next, the expression of transcription factors that regulate the expression of substrate metabolism-related genes was measured in the hearts of CS +/− and WT mice. The mRNA expression levels of peroxisome proliferator-activated receptor γ coactivator 1α (*Pgc1*α) and estrogen-related receptor α (*Err*α), which mainly regulate genes involved in lipid metabolism and mitochondrial electron transport, and that of nuclear respiratory factor 1 (*Nrf1*), which is related to mitochondrial biogenesis, were significantly higher in CS +/− mice than those in WT mice fed an LCKD (Figure 4A). These differences were not observed in the hearts of HFHCD-fed mice (Figure 4B).

### Adenosine Triphosphate Content in the Heart and Gastrocnemius Muscle

**FIGURE 5:**
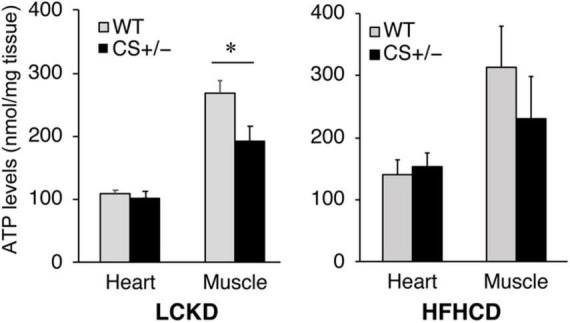
Effects of each diet exposure on the ATP content of the hearts and gastrocnemius muscles of citrate synthase-knockout mice (CS +/−, black bars) and their wild-type siblings (WT, gray bars) fed a LCKD (left panel) or a HFHCKD (right panel). Values are expressed on a wet weight basis (*p < 0.05).

ATP levels were measured in the heart and gastrocnemius muscles of CS +/− and WT mice fed an LCKD or HFHCD. The ATP content in the heart was similar between WT and CS +/− mice in both experiments. However, in the gastrocnemius muscles of LCKD-fed mice, ATP levels were significantly lower in CS +/− mice than that in WT mice (Figure 5). In HFHCD-fed mice, the ATP levels of the gastrocnemius muscle were also lower in CS +/− mice than that in WT mice, but the difference was not significant (Figure 5).

### Glycogen Metabolism in the Muscles of Low-Carbohydrate Ketogenic Diet-Fed Mice

**FIGURE 6:**
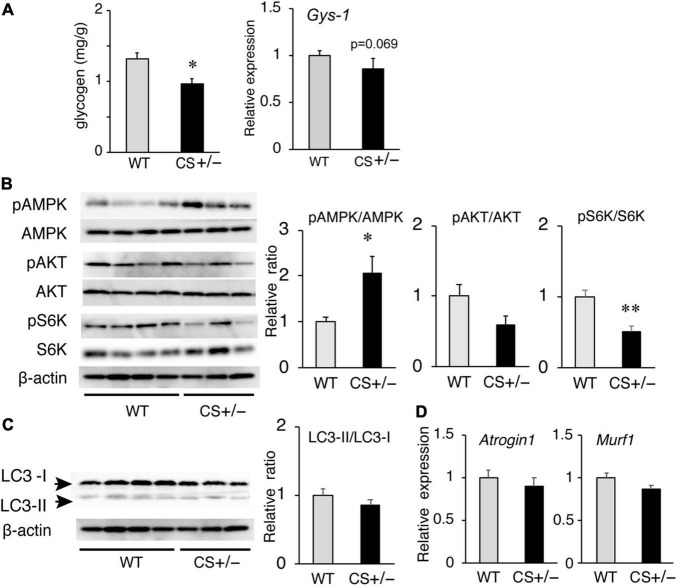
Effects of a ketogenic diet exposure on muscle glycogen, and anabolic and catabolic signaling in the gastrocnemius muscles of citrate synthase-knockout mice (CS +/−) and their wild-type siblings (WT). **(A)** Muscle glycogen content (left panel) and mRNA levels of glycogen synthase 1 (*Gys1*) (right panel). **(B)** Activation of AMP-activated kinase (AMPK) and anabolic signaling molecules Akt and p70 S6 kinase (S6K). β-Actin was used as a loading control. **(C)** Protein levels of autophagy-related proteins LC3-I and LC3-II and the LC3-II/LC3-I ratio. **(D)** mRNA levels of atrogin-1 (*Atg1*) and muscle RING finger-1 (*MuRF-1*). In mRNA and protein measurements, values are expressed as the fold-change compared with the values in WT mice, arbitrarily set to 1 (*p < 0.05; ^**^p < 0.01 vs. WT). The images show representative results of two independent experiments.

Because the muscle ATP levels decreased in LCKD-fed CS +/− mice, we measured the glycogen content, an important energy source for the muscles, in the muscle tissues of LCKD-fed mice. At the end of the experimental period, muscle glycogen storage was significantly decreased in CS +/− mice compared to that in WT mice (Figure 6A). The mRNA levels of glycogen synthase-1 (*Gys-1*), a key enzyme in glycogen synthesis, tended to be lower (*p* = 0.069) in the muscles of CS +/− mice than that of WT mice.

### Anabolic and Catabolic Signaling in the Muscles of Low-Carbohydrate Ketogenic Diet-Fed Mice

We also evaluated the responses of anabolic and catabolic signaling in the muscles of LCKD-fed mice. The phosphorylation of AMP-activated protein kinase (AMPK), a critical cellular ATP sensor, as well as that of protein kinase B (Akt) and p70 S6 kinase (S6K), which are anabolic signaling molecules, was assessed. AMPK phosphorylation was significantly increased in the muscles of CS +/− mice, which is consistent with the decrease in the tissue ATP content (Figure 6B). Phosphorylation of p70 S6K was significantly suppressed in the muscles of CS +/− mice compared with that in WT mice, whereas the levels of phosphorylated Akt did not significantly differ between the two mouse groups (Figure 6B).

To evaluate the catabolic signaling, mRNA levels of the muscle-specific ubiquitin ligases atrogin-1 (*Atg1*), and muscle RING finger-1 (MuRF-1) as well as the levels of light chain 3 proteins LC3-I and LC3-II were determined. CS +/− and WT mice showed no significant differences in the protein ratio of LC3-II to LC3-I (a marker of autophagy) (Figure 6C) or in the mRNA levels of Atg1 and MuRF-1 (Figure 6D), which mediate protein degradation through autophagy-lysosomal and the ubiquitin-proteasome systems, respectively, under conditions of ATP shortage.

### Muscle Morphology of Low-Carbohydrate Ketogenic Diet-Fed Mice

**FIGURE 7:**
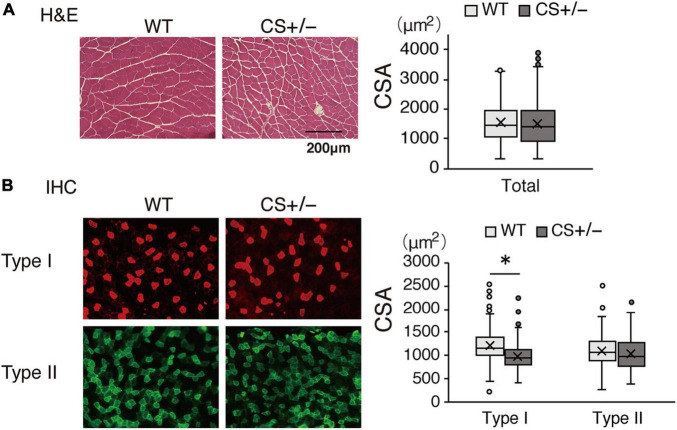
Effects of a ketogenic diet exposure on muscle fiber size. The individual cross-sectional area of **(A)** total fibers stained with hematoxylin and eosin and **(B)** type I and type II fibers stained with immunohistochemistry. Left panel, representative images of the stained sections are shown; right panel, values of the cross-sectional area (CSA) are presented in a box-and-whisker plot. Boxes are constructed with the intervals between the first and third quartiles of the data distribution; lines in the boxes are the median values; positive and negative bars are the 5th and 95th percentile individual values, respectively (*p < 0.05).

In the morphological analysis, CSA of the total myofibers did not differ between WT and CS +/− mice (Figure 7A). However, in LCKD-fed mice, further analysis of different muscle fiber types indicated that the CSA of type I oxidative fibers was significantly lower in CS +/− mice than in WT mice, whereas the CSA of type II glycolytic fibers did not differ between WT and CS +/− mice (Figure 7B).

## Discussion

In this study, we compared the metabolic and physiological responses between WT and CS +/− mice fed with two types of diets: a low-carbohydrate ketogenic diet (LCKD) and a high-fat, high-carbohydrate (HFHCD) diet.

The body weight of mice decreased after starting the LCKD feeding but increased steadily during HFHCD feeding. LCKD consumption mimics the metabolic conditions of long-term starvation, and leads to body weight reduction in both humans and animals (25, 26). The possible causes of this weight loss include appetite loss due to the appetite-suppressant actions of ketosis or changes in appetite control hormones (27, 28), reduced lipogenesis, and increased lipolysis (29, 30). In contrast, HFHCD consumption induces obesity, leading to metabolic conditions such as insulin resistance in rodents (23). However, neither of these diets led to significant differences in body weight changes between CS +/− and WT mice in this study, suggesting that CS insufficiency did not affect hormonal or metabolic factors involved in weight control.

Our results revealed an increase in the cardiac expression levels of genes related to the utilization of energy substrates, including FAs, glucose, lactate, ketone bodies, and amino acids in LCKD-fed CS +/− mice. FAs are the main fuel source for ATP in the heart; the adult myocardium utilizes FAs to supply approximately 60–70% of the total ATP (5). The heart increasingly relies on FA oxidation during intracellular glucose shortages, which occur under certain conditions such as diabetes (31). Thus, in the fasting-mimicking LCKD-fed mice, the increased expression of genes related to FA oxidation in CS +/− mouse hearts may be an adaptive response to energy shortages caused by CS insufficiency.

We also observed increased gene expression of the PGC1α coactivator and its transcription factor binding partners, ERRα and NRF1, in the hearts of CS +/− mice. These molecules amplify mitochondrial FA oxidation and respiration by regulating the expression of genes related to lipid metabolism and oxidative phosphorylation (32, 33). In fact, the cardiac expressions of genes involved in not only FA oxidation but also oxidative phosphorylation were increased in LCKD-fed mice. Therefore, the increase in their expression levels may be part of a compensatory response to CS insufficiency.

It is well known that the shift of substrate utilization from FAs to glucose in the myocardium has been observed in cardiac disorders (34, 35). Nevertheless, unexpectedly, the expression levels of the genes related to cytosolic glycolysis were not altered in the hearts of animals with CS insufficiency. Similarly, recent studies showed that glycolysis and glucose oxidation did not increase in failing hearts (36, 37) and that the heart consumes small amounts of glucose as a fuel source, whether healthy or failing (38). In agreement with these findings, our results suggest that cardiac tissues do not enhance glycolysis as an adaptive strategy to compensate for energy shortages.

Lactate and ketone bodies have not received much attention as oxidative fuel for the heart. However, in humans and rodents, both fuels enter the cardiomyocytes mainly through monocarboxylate transporter-1 (MCT-1) (39) and contribute to energy production as an efficient energy substrate in the heart (40). Our results showed that the expression levels of genes related to lactate and ketone oxidation were significantly increased in the heart of LCKD-fed CS +/− mice. As important energy substrates, the consumption of lactate and ketone bodies increases by nearly two and threefold in failing hearts, respectively (38), and the cardiac expression levels of *Mct1* and *Scot* are upregulated under conditions of energy deficiency such as during exercise training (41) and in the failing heart (42). Therefore, the oxidation of lactate and ketone bodies may be an alternative pathway of energy production in the CS-insufficient heart.

The major amino acids consumed by the heart are glutamate and branched amino acids (BCAAs), which contribute to approximately 5% of total ATP generation (38, 43). Our results revealed increased expression levels of genes related to amino acid catabolism, particularly BCAAs, in the hearts of LCKD-fed CS +/− mice. The contribution of amino acids to energy production in the heart has not been widely examined; however, a recent study revealed that the levels of BCAAs were increased significantly in the heart a few days after pressure overload or surgical infarction (44). Hence, BCAAs may be alternative fuels in energy-deficient hearts. Increased expression levels of genes encoding BCAA catabolic enzymes may be an adaptive compensation in CS-deprived hearts under conditions of a limited glucose supply.

In contrast to the results observed in the heart, the metabolic gene expression levels in the muscles showed no significant differences between CS +/− and WT mice, even in those consuming a LCKD. Thus, the ATP content decreased, and AMPK was activated in the muscles of LCKD-fed CS +/− mice. These findings suggest that the metabolic gene expression in the muscle tissues is not increased as a compensatory response to decreased energy production. The mechanism by which the different adaptive responses to insufficient CS are induced in the heart and the muscles remains unclear. Nonetheless, our findings are consistent with those of a previous study revealing that the ATP content and production were decreased by approximately 50% during aging in the muscle but not in the heart mitochondria (45).

Under restricted carbohydrate intake, we observed that the storage of glycogen, an important fuel for muscle contraction, and the activation of S6K, a key anabolic signaling molecule, were significantly suppressed in the muscles of CS +/− mice. Further, the muscles presented fiber atrophy predominantly in mitochondria-rich type I fiber. Under ATP deprivation, the cells shift from a state of growth to that of survival mode. A previous study reported that increased AMPK activity inhibited mTOR and its downstream targets S6K, resulting in reduced cell sizes and growth rates to protect cells from energy deprivation-induced apoptosis (46). Furthermore, leg muscles release large amounts of amino acids, which act as alternative energy sources for the heart (38). Therefore, the inhibition of glycogen storage and signaling suppression of protein synthesis in the muscles of CS +/− mice may help ensure an adequate fuel supply to the heart when dietary carbohydrates are restricted.

In previous studies, mice fed an HFHCD for 4 months developed heart failure as well as cardiometabolic alterations associated with mitochondrial protein modification (47, 48). Another study showed that HFHCD-feeding led to decreased glucose tolerance in female mice with genetically lower CS activity compared to that in wild-type mice (22). Therefore, we expected that HFHCD-fed CS +/− mice would develop metabolic disarrangement, at least in the heart. However, no significant differences were observed in metabolic parameters in both the heart and gastrocnemius muscles between CS +/− and WT mice. Thus, further studies with longer exposure to HFHCD or using female animals are needed to clarify whether HFHCD consumption affects energy metabolism in the heart and skeletal muscles of CS +/− mice.

This study has some limitations. First, we assessed mRNA expression related to mitochondrial capacity but did not measure mitochondrial content. As the expression of the mitochondrial DNA transcript *Cox-1* was increased in the heart of LCKD-fed mice, CS insufficiency might lead to an increase in not only gene expression but also mitochondrial copy number. This requires further investigation. Second, we did not evaluate the functional changes in the heart of LCKD-fed mice. Considering the increase in the expression of genes related to fuel utilization in these mice, it is necessary to investigate whether the cardiac functions are preserved.

In conclusion, in mice under ketogenic conditions and with low CS activity, the heart showed increased expression of genes related to fuel utilization. In contrast, suppression of anabolic signaling leading to oxidative fiber atrophy was observed in the muscles. These findings suggest that the heart switches between different fuel sources by regulating gene expression to adapt to chronic energy shortages. In contrast, the skeletal muscles may sacrifice their growth to secure fuel sources for the heart. Our data provide an overview of fuel use in organs under conditions of energy shortage and a basis for understanding the etiology of metabolic disorders related to impaired TCA cycle metabolism.

## Data Availability Statement

The raw data supporting the conclusions of this article will be made available by the authors, without undue reservation.

## Ethics Statement

The animal study was reviewed and approved by the Animal Ethics Committee of Ochanomizu University.

## Author Contributions

KS, YH, RT, NW, CO, and YS: investigation and data curation. KS, RT, and KI: formal analysis. KS and KI: writing the manuscript. HS: review and editing the manuscript. KI: conceptualization and project administration. YS and KI: funding acquisition. All authors contributed to the article and approved the submitted version.

## Conflict of Interest

The authors declare that the research was conducted in the absence of any commercial or financial relationships that could be construed as a potential conflict of interest.

## Publisher’s Note

All claims expressed in this article are solely those of the authors and do not necessarily represent those of their affiliated organizations, or those of the publisher, the editors and the reviewers. Any product that may be evaluated in this article, or claim that may be made by its manufacturer, is not guaranteed or endorsed by the publisher.

## Abbreviations

Akt: protein kinase B
AMPK: AMP-activated protein kinase
Atg1: atrogin-1
ATP: adenosine triphosphate
BCAA: branched-chain amino acid
CS: citrate synthase
CSA: cross-sectional area
ERR α: estrogen-related receptor α
FA: fatty acid
Gys-1: glycogen synthase-1
HFHCD: high-fat high-carbohydrate diet
LCKD: low-carbohydrate ketogenic diet
KO: knockout
LC3: light chain 3
MCT-1: monocarboxylate transporter-1
MCT-1: monocarboxylate transporter-1
MuRF-1: muscle RING finger-1
NRF1: nuclear respiratory factor 1
PGC1 α: peroxisome proliferator-activated receptor γ coactivator 1 α
S6K: p70 S6 kinase
TCA: tricarboxylic acid
WT: wild-type.
