# Corrigendum: Citrate synthase insufficiency leads to specific metabolic adaptations in the heart and skeletal muscles upon low-carbohydrate diet feeding in mice

**DOI:** 10.3389/fnut.2023.1193530

**Published:** 2023-04-06

**Authors:** Kanako Sumi, Yuiko Hatanaka, Reina Takahashi, Naoko Wada, Chihiro Ono, Yuri Sakamoto, Hirohito Sone, Kaoruko Iida

**Affiliations:** ^1^Department of Food and Nutrition Science, Graduate School of Humanities and Sciences, Ochanomizu University, Bunkyo, Japan; ^2^Department of Clinical Dietetics and Human Nutrition, Faculty of Pharmacy and Pharmaceutical Sciences, Josai University, Sakado, Japan; ^3^Department of Hematology, Endocrinology and Metabolism, Faculty of Medicine, Niigata University, Niigata, Japan; ^4^Department of Endocrinology and Metabolism, Faculty of Medicine, University of Tsukuba, Tsukuba, Japan; ^5^The Institute for Human Life Innovation, Ochanomizu University, Bunkyo, Japan

**Keywords:** citrate synthase, heart, knockout mice, skeletal muscle, TCA cycle

In the published article, there was an error regarding the affiliations for Kaoruko Iida and Hirohito Sone. As well as having affiliations #1 and #5, or #3, they should also have #4 Department of Endocrinology and Metabolism, Faculty of Medicine, University of Tsukuba, Tsukuba, Japan.

In the published article, there was an error in Materials and Methods. There was a lack of information on how to create CS knockout mice.

A correction has been made to **Materials and Methods**, “*Animals and Rearing Conditions*,” first paragraph. This sentence previously stated:

“Heterozygous CS +/− mice with a C57BL6/J background were kindly provided by Dr. Shimano, University of Tsukuba (Ibaraki, Japan).”

The corrected sentence appears below:

“Heterozygous CS +/− founder mice were created at Lexicon Genetics from their OmniBank library of knockout embryonic stem cell clones. We crossed this CS +/− founder mice more than ten times to transfer the null mutation onto the C57BL6/J genetic background. We used only male mice for the present studies.”

In the published article, there was an omission in Acknowledgments. There must be a reference to the contribution and funding support to the work on the article.

A correction has been made to Acknowledgments section. This section previously stated:

We thank Profs. H. Shimano and N. Yamada for providing the CS+/− mice.

The corrected sentence appears below:

