# Supplementary material for: Citrate Synthase Insufficiency Leads to Specific Metabolic Adaptations in the Heart and Skeletal Muscles Upon Low-Carbohydrate Diet Feeding in Mice
Source: Front Nutr. 2022 Jul 7;9:925908. doi: 10.3389/fnut.2022.925908 (PMC9302927; doi:10.3389/fnut.2022.925908)
Supplement: Supplementary file 1 [file Table_1.DOCX]

Table S1. Ingredient composition of the diets

| **Ingredient（g）** | **KD** | **HFHCD** |
| --- | --- | --- |
| Casein | 19.5 | 20 |
| L-cystine | 0.3 | 0.3 |
| Soybean oil | 6.8 | 1.0 |
| Lard | 64 | 20 |
| Corn starch | 0 | 15 |
| Sucrose | 0 | 34 |
| Cellulose | 4.9 | 5.0 |
| Vitamin mix ^a^ | 1.0 | 1.0 |
| Choline bitartrate | 0.25 | 0.25 |
| Mineral mix ^b^ | 3.4 | 3.5 |
| Total | 100 | 100 |
| Energy [kcal/100 g] | 720.8 | 473.2 |
| Protein [kcal/100g (% of energy)] | 79.2 (11) | 81.2 (17) |
| Fat [kcal/100 g (% of energy)] | 633.6 (88) | 189.0 (40) |
| Carbohydrate [kcal/100g (% of energy)] | 8.0 (1) | 203.0 (43) |

^a^ Containing 97.4% (wt/wt) sucrose.

^b^ Containing 22.1% (wt/wt) sucrose.

The number of the percentage of energy in the parentheses are round off decimal places.
