# Supplementary material for: Citrate Synthase Insufficiency Leads to Specific Metabolic Adaptations in the Heart and Skeletal Muscles Upon Low-Carbohydrate Diet Feeding in Mice
Source: Front Nutr. 2022 Jul 7;9:925908. doi: 10.3389/fnut.2022.925908 (PMC9302927; doi:10.3389/fnut.2022.925908)
Supplement: Supplementary file 2 [file Table_2.DOCX]

Table S2.　 Primer sequences for the quantitative PCR

|  | Forward | Reverse |
| --- | --- | --- |
| *Cs* | TTGGGAGCCAAGAACTCATC | TCTGGCCTGCTCCTTAGGTA |
| *Ogdh* | CAGCTCGCTGTCTGAGTACG | AATGATGCACTGTGCCATGT |
| *Sucla2* | TTGTGCATGGATGCAAAGAT | CTGCTTCTTTGTCCCTTTCG |
| *Glut4* | GACGGACACTCCATCTGTTG | CATAGCTCATGGCTGGAACC |
| *Hk2* | GAAGGGGCTAGGAGCTACCA | CTCGGAGCACACGGAAGTT |
| *Pk* | CGATCTGTGGAGATGCTGAA | AATGGGATCAGATGCAAAGC |
| *Cd36* | TGGCCTTACTTGGGATTGG | CCAGTGTATATGTAGGCTCATCCA |
| *Cpt1b* | GCACACCAGGCAGTAGCTTT | CAGGAGTTGATTCCAGACAGGTA |
| *Mcad* | ACTGACGCCGTTCAGATTTT | GCTTAGTTACACGAGGGTGATG |
| *Ldh* | TGTCTCCAGCAAAGACTACTGT | GACTGTACTTGACAATGTTGGGA |
| *Mct1* | GTGACCATTGTGGAATGCTG | CTCCGCTTTCTGGCCACATCTC |
| *Scot* | TGGCCAACTGGATGATACCTGG | TCCATGGTGACCACCACTTTGG |
| *Glud1* | CTATGGAGCTGGCCAAGAAG | CCTATGGTGCTGGCATAGGT |
| *Bcat2* | TGGAGTGGAATAACAAGGCTG | GTCTCCACCTTTGTATGCTTTC |
| *Bckdha* | CAGATGCCTGTTCACTACGG | CCCTCGCCAAAGTAACAGATC |
| *PGC1α* | CGGAAATCATATCCAACCAG | TGAGGACCGCTAGCAAGTTTG |
| *Pparα* | GAGAATCCACGAAGCCTACC | AATCGGACCTCTGCCTCTTT |
| *Errα* | TTCGCGACTGCAAGCTC | CACAGCCTCAGCATCTTCAATG |
| *Nrf1* | AATGTCCGCAGTGATGTCC | GCCTGAGTTTGTGTTTGCTG |
| *Nrf2* | CTTGGGTTGGTGATGAAGGT | CCGTAATGCACGGCTAAGTT |
| *Cox 1* | TTTTCAGGCTTCACCCTAGATGA | GAAGAATGTTATGTTTACTCCTACGAATATG |
| *Cyt b* | GCCACCTTGACCCGATTCT | TTGCTAGGGCCGCGATAAT |
| *Cyt c* | ACCAAATCTCCACGGTCTGTT | GGATTCTCCAAATACTCCATCAG |
| *Atp5b* | GAGGTCTTCACGGGTCACAT | ATGGGTCCCACCATGTAGAA |
| *Atrogin1* | GCAGAGAGTCGGCAAGTC | CAGGTCGGTGATCGTGAG |
| *Murf1* | CAACCTGTGCCGCAAGTG | CAACCTCGTGCCTACAAGATG |
| *β-actin* | CTAAGGCCAACCGTGAAAAG | ACCAGAGGCATACAGGGACA |

Cs; citrate synthase

Ogdh; oxoglutarate dehydrogenase

Sucla2; succinate-CoA ligase 2

Glut4; glucose transporter 4

Hk2; hexokinase 2

Pk; pyruvate kinase

Cd36; cluster of differentiation 36

Cpt1b; carnitine palmitoyl transferase 1-b

Mcad; medium-chain acyl-CoA dehydrogenase

Ldh; lactate dehydrogenase

Mct1; monocarboxylic acid transporter 1

Scot; succinyl CoA 3-oxoacid CoA transferase

Glud1; glutamate dehydrogenase 1

Bcat2; branched-chain amino transaminase 2

Bckdha; branched chain α-keto acid dehydrogenase E1 alpha

Pgc1α; peroxisome proliferator-activated receptor gamma, coactivator 1 alpha

Pparα; peroxisome proliferator-activated receptor α

Errα; estrogen-related receptor alpha

Nrf; nuclear respiratory factor

Cox 1; cytochrome c oxidase 1

Cyt b; cytochrome b

Cyt c; cytochrome c

Atp5b; ATP synthase F1 subunit beta

Murf1; muscle ring finger 1
