# Supplementary material for: Citrate Synthase Insufficiency Leads to Specific Metabolic Adaptations in the Heart and Skeletal Muscles Upon Low-Carbohydrate Diet Feeding in Mice
Source: Front Nutr. 2022 Jul 7;9:925908. doi: 10.3389/fnut.2022.925908 (PMC9302927; doi:10.3389/fnut.2022.925908)
Supplement: Supplementary file 3 [file Image_1.PDF]

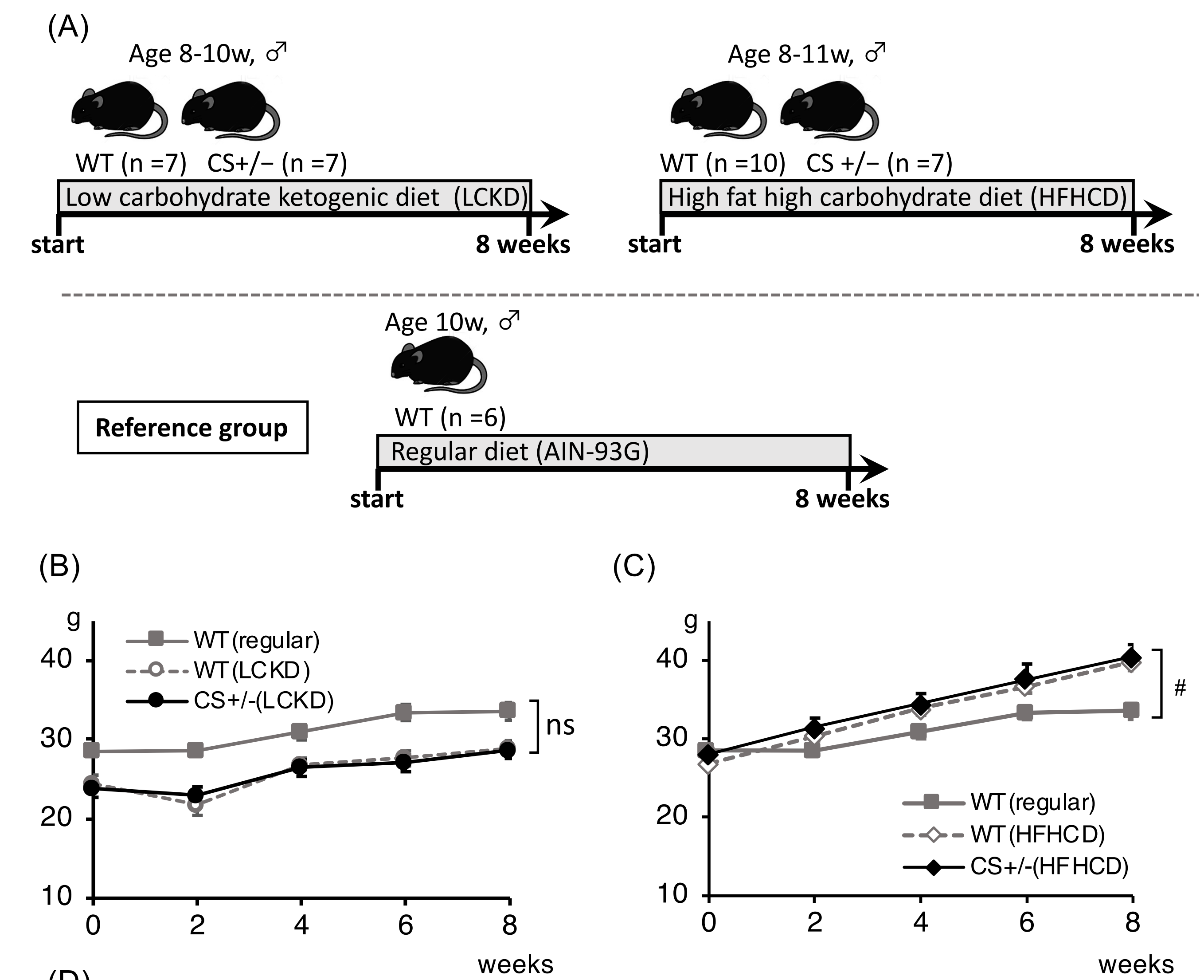

(D)

	LCKD		Regular diet
	WT	CS+/−	WT
Glucose [mg/dL]	234.3 ± 13.4	204.9 ± 14.4	250.8 ± 4.4
Insulin [ng/ml]	0.86 ± 0.16	1.38 ± 0.37	n.d.
Triglyceride [mg/ml]	43.6 ± 2.5	45.0 ± 6.6	34.8 ± 5.2
NEFA [mEq/L]	1.05 ± 0.08**	1.12 ± 0.11**	0.50 ± 0.08
3-HB [μmol/L]	1387.4 ± 292.5**	1320.5 ± 265.6**	174.5 ± 49.3

HFHCD		Regular diet
WT	CS+/−	WT
299.0 ± 14.9*	268.9 ± 11.2	250.8 ± 4.4
3.86 ± 1.34	3.69 ± 0.81	n.d.
51.2 ± 3.2**	56.9 ± 4.9**	34.8 ± 5.2
0.63 ± 0.08	0.80 ± 0.08*	0.50 ± 0.08
235.9 ± 16.3	211.2 ± 24.9	174.5 ± 49.3

Supplementary Figure 1. Comparison of each parameter in mice in the main experiment with that in the reference mice fed a regular diet. (A) Timeline of the experimental protocol. (B) Body weight of hetero knockout mice (CS+/−) and their wild-type siblings (WT) fed an LCKD and that of reference WT mice fed a regular diet, or (C) body weight of CS+/− and WT mice fed an HFHCD and that of reference WT mice fed a regular diet, during the 8-week experimental period. A two-way ANOVA was performed to determine differences among groups. #, the interaction is significant at $p < 0.05$. ns, the interaction is not significant. (D) Blood parameters of mice in each group. For the comparison, a one-way ANOVA followed by Tukey multiple comparison test was performed. *, $p < 0.05$; **, $p < 0.01$ vs. WT fed a regular diet.
